# Effects of Liuzijue Qigong on respiratory function, trunk control, and balance after stroke: a randomized controlled trial

**DOI:** 10.3389/fnagi.2026.1844814

**Published:** 2026-05-28

**Authors:** Chen Wang, He Li, Junwu Yu, Faming Yang, Lili Hou

**Affiliations:** 1 Department of Nursing, Shanghai Ninth People’s Hospital, Shanghai Jiao Tong University School of Medicine, Shanghai, China; 2 School of Physical Education, Shanghai Normal University, Shanghai, China; 3 Ningbo College of Health Sciences, Ningbo, China

**Keywords:** balance, Liuzijue Qigong, respiratory function, stroke, trunk control

## Abstract

**Purpose:**

To evaluate the effects of adding Liuzijue Qigong (LQG) to standard rehabilitation on respiratory function, trunk control, and balance after stroke, and to explore a possible respiratory-core-balance pathway.

**Materials and methods:**

In this assessor-blinded randomized controlled trial, 120 post-stroke inpatients were randomly assigned (1:1) to LQG plus standard rehabilitation or standard rehabilitation alone for 3 weeks. Primary outcomes were trunk control, assessed by the Trunk Impairment Scale (TIS), and pulmonary function, assessed by forced vital capacity (FVC) and peak expiratory flow (PEF). Secondary outcomes included balance, assessed by the Berg Balance Scale (BBS), affected-side external oblique (EO) surface electromyography (sEMG), forced expiratory volume in 1 s (FEV_1_), and the forced expiratory volume in 1 s to forced vital capacity ratio (FEV_1_/FVC). Between-group differences in change scores were analyzed, and exploratory correlation and path analyses were performed.

**Results:**

Both groups showed significant within-group improvements in the TIS and BBS after the 3-week intervention. However, between-group differences in TIS and BBS change scores were not statistically significant (*p* = 0.092 and *p* = 0.073, respectively). Compared with the control group, the LQG group showed greater improvements in FVC (Δ0.51 vs. 0.25 L; *p* < 0.001), PEF (Δ52.0 vs. 11.0 L/min; *p* < 0.001), and affected-side EO activation (Δ11.0 vs. 3.0; *p* < 0.001). In exploratory analyses, change scores were positively correlated (ΔPEF–ΔEO *r* = 0.58; ΔEO–ΔTIS *r* = 0.63; ΔTIS–ΔBBS *r* = 0.50; all *p* < 0.001), and the path model was consistent with a possible sequential respiratory-core-balance pathway.

**Conclusion:**

Adding LQG to standard rehabilitation yielded greater improvements in FVC, PEF, and affected-side EO activation after stroke. Although additional gains in trunk control and balance scales were not statistically significant over 3 weeks, exploratory analyses suggested a possible respiratory-core-balance pathway that warrants further validation.

**Trial registration:**

https://www.chictr.org.cn/. Identifier (ChiCTR1800020170).

## Introduction

Post-stroke balance dysfunction increases the risk of falls and reduces quality of life (Liu et al., 2025). Conventional rehabilitation after stroke has mainly focused on restoring limb motor function. In contrast, the contribution of core muscles, especially the diaphragm and abdominal muscles, has received less attention. Emerging evidence suggests that trunk muscles are involved not only in respiration but also in postural control (Hodges and Gandevia, 2000; Hodges et al., 2001). Hodges and colleagues reported that the diaphragm shows tonic activation during limb movement and acts synergistically with core muscles such as the transversus abdominis (TrA) to increase intra-abdominal pressure (IAP), thereby contributing to feedforward stabilization of the spine (Hodges and Gandevia, 2000). Consistent with this view, diaphragm-related functional parameters, such as thickness and excursion, have been associated with balance performance (Kocjan et al., 2018). When ventilatory demand increases or neural control is impaired, as after stroke, the postural contribution of the diaphragm may be reduced, which may in turn compromise postural control (Hodges et al., 2001; Yu et al., 2021). These findings suggest that integrating respiratory training into trunk rehabilitation may be a useful strategy for improving balance after stroke.

Liuzijue Qigong (LQG) is a traditional Chinese mind–body exercise that combines specific phonation mouth shapes (e.g.,“Xu” and “He”) with reverse abdominal breathing (RAB), forming a coordinated pattern of phonation, breathing, and movement (Wang et al., 2020; Wang et al., 2021). Unlike conventional breathing exercises, LQG incorporates vocalization during expiration. This pattern may moderately narrow the glottis and increase expiratory airflow resistance (Wang et al., 2021). The RAB-based strategy resembles the maximal abdominal contraction maneuver (MACM) and may facilitate concentric activation of abdominal core muscles, such as the TrA and external oblique (EO), thereby increasing IAP and improving lumbopelvic stability (Kwon et al., 2021). Previous studies have shown that LQG can improve trunk postural control and balance after stroke, and some reports suggest that it may be more effective than core stabilization training alone (Wang et al., 2020; Zhang et al., 2022). LQG has also been associated with improved respiratory function (Zeng et al., 2025). However, most previous studies have relied mainly on clinical rating scales, such as the Berg Balance Scale (BBS) and the Trunk Impairment Scale (TIS), and have provided limited objective neurophysiological evidence from measures such as surface electromyography (sEMG). As a result, it remains unclear how this phonation-guided expiratory pattern influences activation of the paretic-side core muscles and how such changes may relate to trunk control and balance.

Based on the biomechanical role of IAP, we hypothesized that LQG may enhance coordinated contraction of the respiratory muscles and abdominal wall through its characteristic reverse abdominal breathing pattern, thereby increasing IAP and improving lumbopelvic stability (Hodges and Gandevia, 2000; Cholewicki et al., 1999). However, clinical scales such as the BBS and TIS may not fully capture neuromuscular adaptations. In addition, sEMG cannot directly quantify the activity of deep core muscles such as the TrA. For this reason, we selected the EO for assessment. The EO contributes to active expiration, participates in IAP modulation, and helps stabilize the trunk and pelvis during postural tasks, especially during anti-rotation control (Kawabata and Shima, 2023; Drysdale et al., 2004). Accordingly, EO sEMG activity was used as an index of changes in core recruitment strategies associated with altered breathing patterns. To date, randomized controlled evidence in post-stroke populations remains limited for LQG interventions that quantify abdominal muscle recruitment using sEMG. Moreover, neurophysiological evidence linking respiratory muscle function to improvement in balance remains scarce (Kocjan et al., 2018).

In this study, we examined the effects of LQG on respiratory and trunk-related outcomes in individuals after stroke. In addition to clinical outcomes, we collected objective physiological data, including sEMG, and performed mediation modeling to explore a potential mechanistic pathway. Specifically, we tested a sequential pathway in which improved respiratory function was associated with greater activation of the paretic-side EO, followed by better trunk control and, subsequently, better balance performance. Through this analysis, we aimed to determine whether the proposed pathway was supported by the data and to estimate the extent to which these intermediate variables accounted for the overall association, thereby providing evidence to inform the integration of respiratory training into trunk rehabilitation.

## Materials and methods

### Study design, setting, and participants

The study protocol was approved by the Ethics Committee of Shanghai Xuhui Central Hospital (Approval No. 2018044) and was conducted in strict accordance with the institutional requirements and the pre-specified research plan. All participants provided written informed consent prior to inclusion. This trial was conducted and reported in adherence to the CONSORT (Consolidated Standards of Reporting Trials) guidelines. Due to institutional policies and the terms of informed consent, the raw dataset generated during the current study is not publicly available, but may be available from the first and corresponding authors upon reasonable request. The trial was registered with the Chinese Clinical Trial Registry (Trial ID: ChiCTR1800020170).

### Participants

Participants were inpatients with post-stroke hemiplegia recruited from Shanghai Xuhui Central Hospital between December 2018 and December 2021, with a total of 120 individuals enrolled. Inclusion criteria were: (1) diagnosis of stroke confirmed by Computed Tomography (CT) or Magnetic Resonance Imaging (MRI); (2) time since onset ≤6 months (recovery stage); (3) stable vital signs; (4) clear consciousness and preserved cognitive function with a Mini-Mental State Examination (MMSE) score ≥2; and (5) provision of written informed consent. Exclusion criteria were: (1) severe primary dysfunction of the heart, lung, liver, or kidney; (2) history of severe respiratory disease; (3) severe osteoporosis or fracture preventing completion of the training movements; and (4) severe aphasia or cognitive impairment that precluded cooperation with assessments and training.

### Study design

This study was a single-center, parallel-group, randomized controlled trial with assessor-blinded outcome evaluations. The trial was conducted at Shanghai Xuhui Central Hospital. Eligible participants underwent baseline assessments and received standard education regarding physical activity recommendations post-stroke. Participants were randomly assigned to either the LQG or the CG. Follow-up outcome evaluations (post-test) were performed at the end of the 3-week intervention. Participant enrollment commenced in December 2018, and data collection was concluded in December 2021. This study was conducted as part of the first author’s master’s research project at Shanghai Xuhui Central Hospital. During the study period, the first author participated in study implementation at the trial site. At the time of manuscript submission, the listed affiliations reflected the authors’ current institutional affiliations.

### Randomization and data collection

Following baseline assessments, participants were randomly assigned to either the control group (CG) (conventional rehabilitation treatment) or the intervention group (conventional rehabilitation treatment plus LQG) in a 1:1 ratio. The allocation sequence was generated using a random number table before group assignment and was implemented after completion of baseline assessment. All outcome measures were evaluated using standardized protocols by trained assessors who remained blinded to group allocation throughout the study. All tests were conducted in a consistent manner at designated time points and locations. No formal allocation concealment procedure was implemented in the original study protocol, and this should be considered when interpreting the risk of selection bias.

### Interventions

#### Standard care

Standard care (SC) consisted of comprehensive post-stroke rehabilitation, which included: (1) Physical Therapy: Incorporating neurodevelopmental facilitation (Bobath concept), limb function training, and routine core stabilization exercises (e.g., bridging, trunk flexion/extension); (2) Respiratory Management: Consisting of basic pursed-lip breathing and diaphragmatic breathing instructions to prevent pulmonary complications; and (3) Occupational Therapy (Zhang et al., 2022). The treatment was administered for 30 min per session, once daily, five times per week for 3 weeks (totaling 15 sessions) (Wang et al., 2022).

### Liuzijue Qigong intervention

In addition to SC, the intervention group participated in LQG for 20 min per session, delivered on the same schedule as SC (once daily, 5 days/week for 3 weeks; 15 sessions) (Wang et al., 2022). The protocol consisted of six specific vocalizations (“Xu”, “He”, “Hu”, “Si”, “Chui”, and “Xi”) synchronized with specialized guiding movements (Wang et al., 2022). Training emphasized slow, gentle nasal inhalation followed by prolonged oral exhalation (Zhang et al., 2022). To ensure intervention fidelity, all therapists followed a standardized LQG protocol and delivered training using standardized verbal cues and unified audio/video guidance to maintain consistent pacing. Participants were instructed to adopt an “inhalation shorter than exhalation” rhythm during phonation-guided expiration. Movements were adapted based on each participant’s trunk control and balance status, with therapists providing manual assistance or facilitating unaffected-side synergy when necessary.

### Safety monitoring and adverse events

All sessions were supervised by certified therapists who monitored vital signs and tolerance. Training was terminated immediately if symptoms occurred, such as dizziness, chest pain, or postural instability. Any adverse events (AEs) were recorded and managed according to standardized protocols. Serious AEs were reported to the Principal Investigator and the Ethics Committee within 24 hours (Wang et al., 2020).

### Intervention adherence

Attendance was recorded for each session using standardized logs. Adherence was defined as the number (and percentage) of completed sessions out of the planned 15 sessions. Missed sessions and reasons for absence (if available) were documented.

### Outcome measures

All outcomes were assessed at baseline (pre-intervention) and after the 3-week intervention by evaluators trained and tested for interrater consistency. The primary outcomes were trunk control, assessed using the TIS, and pulmonary function, with forced vital capacity (FVC) and peak expiratory flow (PEF) specified as the primary pulmonary function outcomes. Additional pulmonary function indicators included forced expiratory volume in 1 s (FEV_1_) and the ratio of forced expiratory volume in 1 s to forced vital capacity (FEV_1_/FVC). Secondary outcomes included balance, assessed using the BBS, and trunk muscle sEMG, with paretic-side EO activity as the primary sEMG indicator. Assessments were scheduled within the same or adjacent time window, and participants rested for ≥10 min before each session.

Trunk control, balance, motor function, and activities of daily living.

Trunk control was evaluated using the TIS, which includes static sitting balance, dynamic sitting balance, and coordination (total score 0–23; higher scores indicate better trunk control) (Verheyden et al., 2004). The TIS was administered in sitting with foot support according to standardized procedures. Balance was assessed using the BBS (14 items scored 0–4, total score 0–56; higher scores reflect better balance) (Giné-Garriga et al., 2010). In the present study, the BBS was used as a clinical measure of balance and as an indirect proxy for fall-risk-related functional status, rather than a direct measure of falls or fall incidence. Motor recovery was measured using the motor domain of the Fugl-Meyer Assessment (FMA), including upper-extremity (0–66) and lower-extremity (0–34) sub-scores (total motor score 0–100; higher scores indicate better motor function) (Sullivan et al., 2011). Activities of daily living (ADL) were assessed using the modified barthel index (MBI), commonly reported on a 0–100 scale, with higher scores indicating greater ADL independence (Shah et al., 1989).

Surface electromyography.

Bilateral sEMG signals were recorded from the rectus abdominis (RA), EO, and lumbar erector spinae (ES). Skin preparation (shaving when necessary, alcohol cleansing, and gentle abrasion) was performed to reduce skin impedance. Bipolar surface electrodes were used with an inter-electrode distance of approximately 2 cm. Electrode locations followed commonly used trunk sEMG placement approaches: ES approximately 3 cm lateral to the L3 spinous process; RA approximately 5 cm superior and 3 cm lateral to the umbilicus; and EO approximately 15 cm lateral to the umbilicus near the midpoint between the anterior superior iliac spine and the lower rib margin, with electrode orientation aligned with muscle fiber direction (Jaiswal et al., 2024).

Given potential weakness in people after stroke and prior studies discouraging maximal voluntary contraction (MVC)-based normalization in this population, a static sitting reference segment (STAT) was used for normalization (Haas et al., 2022). Participants sat on a backless chair/treatment plinth with hips and knees flexed at approximately 90°, feet flat on the floor, and trunk upright; a 15-s STAT segment was recorded. Participants then performed a seated selective trunk lateral flexion/lateral displacement task driven by the rib cage while keeping the pelvis as stable as possible. Movements were performed to the left and right, five repetitions per side, with sufficient rest between repetitions to minimize fatigue effects (Haas et al., 2022). Testing was terminated immediately if dizziness, marked dyspnea, chest discomfort/pain, palpitations, abnormal blood pressure fluctuations, or postural instability occurred; events were documented and managed per protocol.

Sampling frequency was set at ≥1,000 Hz. After band-pass filtering, the root mean square (RMS) was calculated and the peak/maximal sEMG activity during task epochs was extracted. The maximal values from the five repetitions were averaged to obtain a representative measure for each side. Task maxima were normalized to STAT to generate relative values for between-group and pre–post comparisons (Haas et al., 2022).

### Pulmonary function testing

Spirometry was performed in sitting with a nose clip and an airtight seal around the mouthpiece. Participants inhaled maximally to near total lung capacity and then performed a rapid, sustained forced expiration to end-exhalation to obtain forced vital capacity (FVC), forced expiratory volume in 1 s (FEV_1_), and peak expiratory flow (PEF). Quality control followed American Thoracic Society/European Respiratory Society (ATS/ERS) technical standards: at least three acceptable maneuvers were obtained, and the best/maximal value was recorded once repeatability criteria were met (Miller et al., 2005; Graham et al., 2019). Adequate rest was provided between trials, and the test was paused or terminated if dizziness, chest discomfort, or marked fatigue occurred.

### Statistical analysis

Sample size was calculated using G*Power (version 3.1.9.7; Heinrich Heine University Düsseldorf, Düsseldorf, Germany) (Faul et al., 2007; Faul et al., 2009). Sample size planning was informed by G*Power together with local pilot/clinical experience focused on balance-related outcomes and feasibility considerations at the study site. Because directly comparable prior data for the current combination of co-primary outcomes (TIS, FVC, and PEF), as well as EO sEMG, were limited, a 2-sided test was assumed with *α* = 0.05, power = 0.80, and a 1:1 allocation ratio. The minimum planned sample size was 106 participants (53 per group). To accommodate potential non-enrollment and withdrawal during the study, we initially planned to screen about 160 candidates. Ultimately, a total of 120 participants were randomized and successfully included in the final analysis, which exceeded the prespecified minimum sample size.

All statistical analyses and data visualization were performed using R software (version 4.2.0; R Foundation for Statistical Computing, Vienna, Austria) (Rosseel, 2012). The normality of the data distribution was assessed using the Shapiro–Wilk test. Continuous variables were expressed as mean ± standard deviation (SD) if normally distributed, or as median (interquartile range, IQR) if non-normally distributed. Categorical variables were presented as frequencies and percentages [*n* (%)]. For comparisons of baseline characteristics and change scores (*Δ*) between groups, the independent sample *t*-test was used for normally distributed data, whereas the Mann–Whitney *U* test was employed for non-normally distributed data. Within-group comparisons (pre- vs. post-intervention) were analyzed using the paired *t*-test or Wilcoxon signed-rank test, depending on the data distribution. To explore the sequential associations among improvement indicators, Spearman’s rank correlation analysis was performed using R to examine the relationships among ΔPEF, affected ΔEO, ΔTIS, and ΔBBS. Based on these results, a chain mediation model was constructed in R as an exploratory analysis. The model used group as the independent variable, ΔBBS as the dependent variable, and ΔPEF, ΔEO, and ΔTIS as sequential mediators. The significance of path coefficients (*β*) and mediation effects was assessed using the bias-corrected percentile bootstrap method with 5,000 resamples (95% confidence interval [CI]). A two-sided *p* < 0.05 was considered statistically significant. It is important to note that this model provides statistical support for the hypothesized pathway, but does not establish causality.

## Results

**Figure 1:**
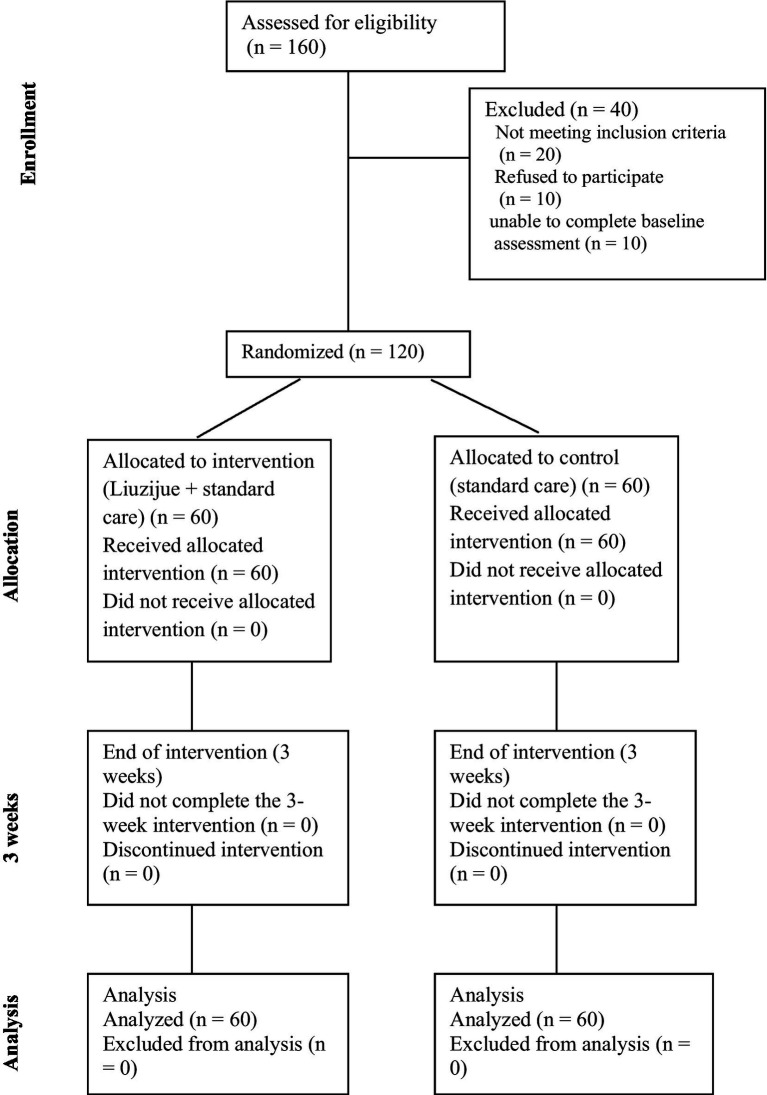
CONSORT flow diagram of participant screening, randomization, allocation, follow-up, and analysis. LQG, Liuzijue Qigong.

Table 1 summarizes the baseline demographic and clinical characteristics of the participants. No statistically significant between-group differences were observed in age, time since onset, height, weight, body mass index (BMI), sex, affected side, diagnosis, or Brunnstrom stage (all *p* > 0.05), indicating comparable baseline characteristics between groups. As shown in Figure 1, 160 participants were screened, 40 were excluded (20 did not meet the inclusion criteria, 10 withdrew, and 10 did not complete baseline assessments), and 120 were randomized and included in the final analysis.

**Table 1:** Baseline characteristics of the participants.

Variables	LQG (*n* = 60)	CG (*n* = 60)	*p*-values
Age (years)	62.28 ± 10.20	61.88 ± 11.10	0.84
Duration of disease (days)	69.88 ± 51.70	69.27 ± 49.78	0.95
Height (cm)	170.37 ± 7.95	168.93 ± 8.36	0.34
Weight (kg)	69.12 ± 8.02	69.42 ± 10.38	0.86
BMI (kg/m^2^)	23.82 ± 2.41	24.39 ± 3.81	0.33
Gender (male/female)	15/45	17/43	0.84
Hemiparesis side (Right/Left)	28/32	26/34	0.85
Diagnosis (Hemorrhage/Infarction)	12/48	14/46	0.83
Brunnstrom stage [*n* (%)]			0.94
Stage I	3 (5%)	4 (6.7%)	
Stage II	15 (25%)	10 (16.7%)	
Stage III	13 (21.7%)	17 (28.3%)	
Stage IV	16 (26.7%)	18 (30%)	
Stage V	10 (16.7%)	9 (15%)	
Stage VI	3 (5%)	2 (3.3%)	

LQG, Liuzijue Qigong; CG, Control Group.

As shown in Table 2, both groups demonstrated significant within-group improvements in the BBS, TIS, FMA, and MBI after the 3-week intervention (*p* < 0.001). However, no statistically significant between-group differences were observed in change scores for BBS, TIS, FMA, or MBI (all *p* > 0.05). Specifically, the between-group *p*-values were 0.073 for BBS and 0.092 for TIS.

**Table 2:** Changes in balance, trunk control, motor function, and activities of daily living before and after intervention in the two groups.

Variables	Group	Pre	Post	Change-values	*p*-values
Berg	LQG	36.0 (32.0, 40.0)	43.0 (36.8, 49.0)^**^	8.0 (3.0, 11.0)	0.073
	CG	36.5 (32.0, 41.0)	42.0 (38.0, 46.0)^**^	5.0 (2.0, 9.0)	
TIS	LQG	12.0 (11.0, 13.0)	14.0 (13.0, 16.0)^**^	2.0 (1.0, 4.0)	0.092
	CG	12.0 (11.0, 13.0)	14.0 (12.0, 15.3)^**^	2.0 (1.0, 3.0)	
FMA	LQG	53.5 (50.0, 58.0)	60.0 (54.0, 67.0)^**^	6.5 (3.8, 9.0)	0.22
	CG	56.0 (49.8, 61.0)	60.0 (55.0, 68.3)^**^	5.0 (3.0, 9.0)	
MBI	LQG	60.0 (55.0, 65.0)	65.0 (60.0, 75.0)^**^	10.0 (3.8, 15.0)	0.70
	CG	60.0 (55.0, 65.0)	65.0 (60.0, 75.0)^**^	10.0 (5.0, 15.0)	

^**^p < 0.01 for within-group comparison before and after intervention; Berg, Berg balance scale; TIS, Trunk impairment scale; FMA, Fugl-Meyer Assessment; MBI, Modified barthel index; ADL, Activities of daily living; LQG, Liuzijue Qigong; CG, Control group.

As shown in Table 3, both groups demonstrated significant within-group increases in FVC and PEF after the intervention. FEV_1_ increased significantly within the LQG (*p* < 0.01), whereas the within-group change in FEV_1_ was not statistically significant in the CG (*p* > 0.05). In between-group comparisons of change scores, the LQG showed significantly greater improvements in FVC (Δ0.51 vs. 0.25 L; *p* < 0.001) and PEF (Δ52.0 vs. 11.0 L/min; *p* < 0.001) than the CG. No significant between-group difference was observed for FEV_1_ (*p* > 0.05). The FEV_1_/FVC decreased in both groups, with a larger reduction in the LQG (*p* < 0.001).

**Table 3:** Changes in pulmonary function before and after treatment in the two groups.

Variables	Group	Pre	Post	Change-values	*p*-values
FVC (L)	LQG	2.91 (2.61, 3.42)	3.51 (3.11, 4.04)^**^	0.51 (0.33, 0.70)^‡‡^	<0.001
	CG	2.87 (2.53, 3.25)	3.16 (2.81, 3.51)^**^	0.25 (0.13, 0.44)	
FEV_1_ (L)	LQG	2.37 (2.12, 2.76)	2.51 (2.21, 2.87)^**^	0.14 (0.05, 0.22)	0.99
	CG	2.27 (2.05, 2.78)	2.39 (2.13, 2.88)	0.13 (0.04, 0.24)	
PEF (L/min)	LQG	272 (231, 335)	322 (278, 401)^**^	52.0 (42.0, 67.3)^‡‡^	<0.001
	CG	297 (233, 344)	308 (242, 353)^**^	11.0 (5.8, 18.0)	
FEV_1_/FVC (%)	LQG	81.3 (78.0, 84.8)	75.1 (70.6, 79.3)^**^	−5.7 (−8.5, −2.5)^‡‡^	<0.001
	CG	81.7 (77.9, 86.9)	79.9 (76.2, 83.5)^**^	−1.1 (−4.2, 2.3)	

^**^p < 0.01 for within-group comparison before and after intervention; ^‡‡^*p* < 0.01 for between-group comparison of change scores. FVC: Forced vital capacity; FEV_1_: Forced expiratory volume in 1 s; PEF, Peak expiratory flow; FEV_1_/FVC: the ratio of FEV_1_ to FVC; LQG, Liuzijue Qigong; CG, Control group.

As shown in Table 4, both groups demonstrated significant within-group increases in sEMG amplitudes after 3 weeks of intervention (*p* < 0.001). In between-group comparisons of change scores, the LQG showed significantly greater improvements in the affected-side EO and RA than the CG (both *p* < 0.001). No significant between-group differences were observed for the unaffected-side EO, unaffected-side RA, or bilateral ES measures (all *p* > 0.05).

**Table 4:** Changes in bilateral EO, ES, and RA sEMG activity before and after intervention in the two groups.

Muscle (Side)	Group	Pre	Post	Change-values	P-values
EO (Aff)	LQG	24.5 (19.8, 29.0)	35.5 (30.8, 42.0)^**^	11.0 (9.0, 14.3)^‡‡^	<0.001
	CG	26.5 (21.8, 33.0)	29.5 (24.0, 36.0)^**^	3.0 (0.0, 5.0)	
EO (Unaff)	LQG	49.0 (40.0, 57.0)	49.0 (42.0, 59.3)^**^	1.0 (0.0, 2.0)	0.892
	CG	49.5 (41.0, 57.5)	51.0 (43.8, 59.3)^**^	1.0 (0.0, 3.0)	
RA (Aff)	LQG	25.0 (22.0, 31.0)	32.5 (27.0, 37.0)^**^	7.0 (4.0, 9.0)^‡‡^	<0.001
	CG	27.0 (20.8, 31.0)	31.0 (25.0, 34.3)^**^	4.0 (2.0, 6.0)	
RA (Unaff)	LQG	38.5 (33.0, 43.0)	39.0 (34.0, 44.0)^**^	1.0 (0.0, 2.0)	0.863
	CG	40.5 (35.8, 46.3)	42.0 (37.0, 47.3)^**^	1.0 (0.0, 3.0)	
ES (Aff)	LQG	45.0 (36.0, 49.5)	46.0 (37.8, 52.0)^**^	1.0 (0.0, 2.3)	0.826
	CG	47.0 (39.8, 52.3)	49.0 (40.0, 54.0)^**^	1.0 (0.0, 3.0)	
ES (Unaff)	LQG	24.0 (20.0, 29.0)	29.0 (24.0, 33.0)^**^	4.0 (3.0, 6.0)	0.072
	CG	23.5 (19.0, 30.0)	26.5 (22.8, 32.3)^**^	3.0 (2.0, 5.0)	

^**^p < 0.01 for within-group comparison; ^‡‡^*p* < 0.01 for between-group comparison of change scores. sEMG, surface electromyography; EO, External oblique; RA, Rectus abdominis; ES, Erector spinae; Aff, Affected side; Unaff, Unaffected side; LQG, Liuzijue Qigong; CG, Control group.

**Figure 2:**
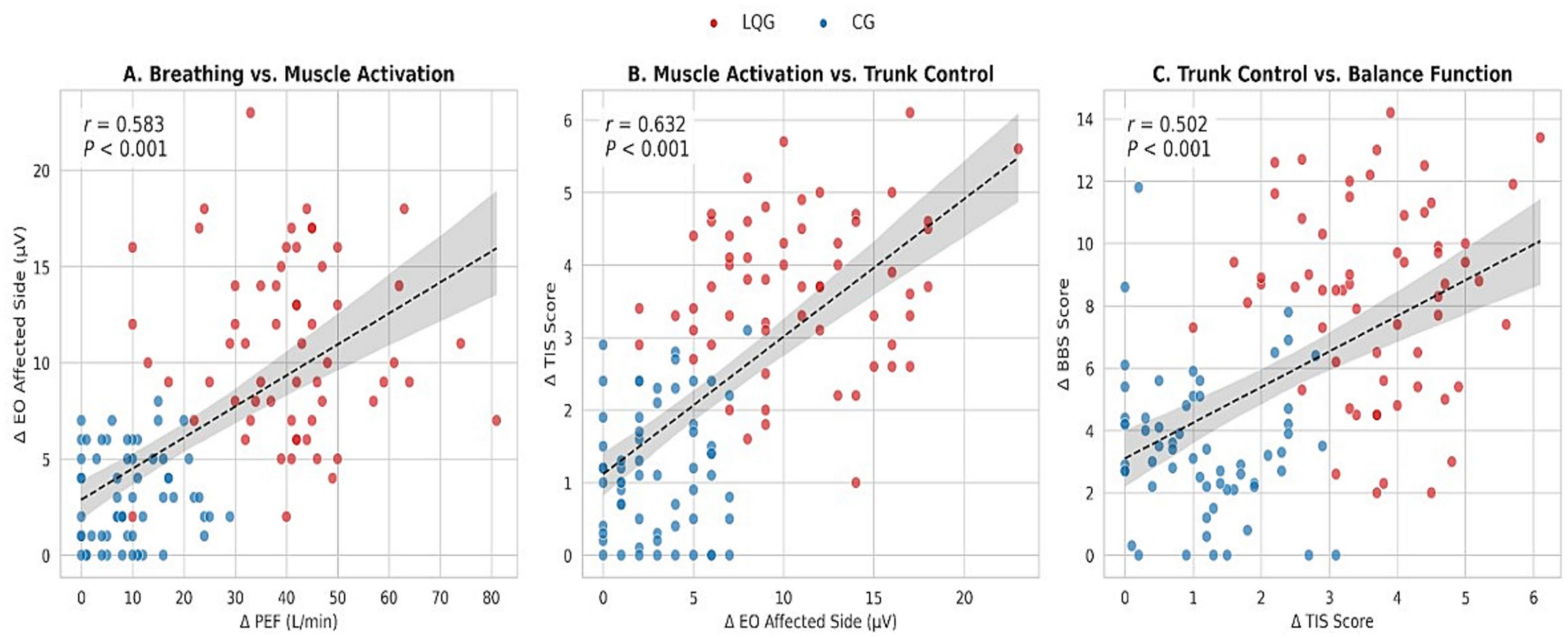
Scatter plots illustrating the correlations between changes (*Δ*) in clinical outcomes. **(A)** Correlation between the change in Peak Expiratory Flow (ΔPEF) and the change in External Oblique activation on the affected side (ΔEO). **(B)** Correlation between ΔEO and the change in Trunk Impairment Scale score (ΔTIS). **(C)** Correlation between ΔTIS and the change in Berg Balance Scale score (ΔBBS). Red dots represent the LQG group; blue dots represent the Control Group (CG). The regression line is shown for visualization only; correlation coefficients were calculated using Spearman’s rank method. Spearman correlation coefficients (r) and *p*-values are presented in the top-left corner of each panel.

**Figure 3:**
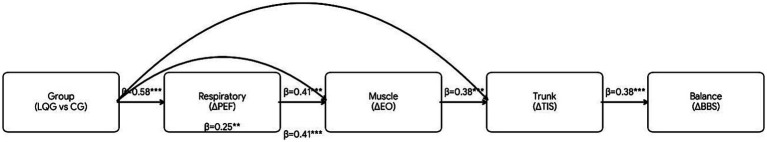
Path analysis of the chain mediation model linking the intervention to balance function. The model illustrates the sequential effects of LQG on respiratory function, muscle activation, trunk control, and balance. Solid arrows indicate significant paths. Values on arrows represent standardized path coefficients (*β*). *p* < 0.01, *p* < 0.001.

In exploratory analyses (Figures 2, 3), significant positive correlations were observed between ΔPEF and affected-side ΔEO, between affected-side ΔEO and ΔTIS, and between ΔTIS and ΔBBS (all *p* < 0.001). ΔPEF was moderately correlated with affected-side ΔEO (*r* = 0.58), affected-side ΔEO was positively correlated with ΔTIS (*r* = 0.63), and ΔTIS was positively correlated with ΔBBS (*r* = 0.50). In exploratory path analysis, the model was consistent with a possible sequential respiratory-core-balance pathway. Group assignment was positively associated with improvement in respiratory function (*β* = 0.58), respiratory improvement was associated with greater EO activation (*β* = 0.41), EO activation was associated with better trunk control (*β* = 0.38), and trunk control was associated with better balance function (*β* = 0.38). A direct association between group assignment and EO activation (*β* = 0.41) was also observed. All reported path coefficients were significant (*p* < 0.01 or *p* < 0.001).

## Discussion

This study found that both groups improved after the 3-week intervention. However, the between-group differences in trunk control and balance-related outcomes, as measured by the TIS and BBS, did not reach statistical significance. Several factors may help explain this finding. The intervention period may have been sufficient to induce physiological changes, but too short for these changes to be fully reflected in broader functional scales. In addition, both groups received active standard rehabilitation, which likely improved trunk control and balance in both groups and reduced the likelihood of detecting an additional between-group effect over a short period. Because the BBS and TIS are composite clinical scales, they may also be less sensitive to short-term neuromuscular adaptations than objective physiological measures. Although the study met its planned sample size, it may still have been better positioned to detect physiological differences than modest differences in complex functional outcomes.

Against this background, the clearer between-group differences in pulmonary function and sEMG remain informative. Previous studies have suggested that LQG could improve balance, trunk control, and respiratory-related indices in stroke patients. In the present study, significant within-group improvements in TIS and BBS were observed in both groups after 3 weeks, consistent with the findings of previous study (Zhang et al., 2022; Zheng et al., 2021); however, the between-group differences in total scale scores did not reach statistical significance. These findings should therefore not be interpreted as showing a clear additional benefit of LQG on balance or trunk control over 3 weeks. The greater gains in FVC, PEF, and sEMG activation of the EO instead suggest that LQG may act earlier at a physiological level. Broader changes on composite clinical scales may require more time to emerge. This pattern suggests that the additional effects of LQG may be captured more readily by physiological indicators than by broader functional scales.

The present findings more directly support a physiological interpretation centered on respiratory improvement and abdominal wall recruitment. Compared with the CG, the LQG showed greater improvements in FVC and PEF, together with greater increases in affected-side EO and RA activation. The positive association between ΔPEF and ΔEO further supports a closer link between improved expiratory performance and abdominal wall recruitment. In practical terms, the breathing pattern required during LQG may increase expiratory load and thereby increase the demand for synergistic contraction of the abdominal wall muscles during breathing and postural tasks (Hodges and Gandevia, 2000; Kawabata and Shima, 2023). This interpretation is more directly supported by the present data than broader neural or autonomic hypotheses.

At the same time, these mechanistic inferences should remain cautious. sEMG of the EO provides only a measurable superficial window of abdominal wall activity. It should not be interpreted as a direct measure of deep core muscle activation, nor as a direct measure of IAP. Even so, the observed increase in EO activity may still be understood within the abdominal bracing or pressurized canister framework. Coordinated activation of the diaphragm and abdominal wall can increase trunk stiffness and mechanical support for the spine (Cholewicki et al., 1999). Prior work has also linked EO activation to IAP regulation during abdominal bracing tasks (Tayashiki et al., 2016). In this sense, the higher EO activation observed here may reflect stronger abdominal wall co-contraction relevant to trunk stabilization. Direct physiological confirmation, however, would require direct monitoring of IAP or diaphragm-abdominal co-activation.

The exploratory path model was also consistent with a sequential pathway linking respiratory improvement, EO activation, trunk control, and balance. This result should be interpreted conservatively. The model provides statistical support for a hypothesized pathway, but it does not establish physiological causality. The observed direct effects also suggest that the association between LQG and clinical outcomes may not be explained entirely by the respiratory pathway and may involve additional factors, including postural training effects, other neuromuscular adjustments (Maeo et al., 2013; Oh et al., 2020), attentional engagement, or psychological influences. By contrast, explanations involving semi-occluded vocal tract mechanisms (Titze, 2009), cortical motor networks (Belyk et al., 2021; Nierat et al., 2015), or autonomic regulation (Larson et al., 2020; Meehan and Shaffer, 2024) were not directly examined in the present study. These higher-level mechanisms are therefore better regarded as literature-based speculations and topics for future research rather than conclusions supported directly by the current data.

Both groups improved after the intervention in balance- and trunk-related outcomes. Some of these changes were within the range of minimal clinically important differences (MCIDs) reported in the literature (Tamura et al., 2022; Hiengkaew et al., 2012; Ishiwatari et al., 2023). This suggests that rehabilitation training itself, including SC, is beneficial during stroke recovery. However, the between-group differences in BBS and TIS were not statistically significant. The present study therefore does not support a clear additional clinical benefit of LQG in these scale-based outcomes over 3 weeks. The clearer between-group differences in pulmonary function and sEMG suggest that LQG may contribute through different physiological pathways. Whether these physiological gains can be translated into additional clinical balance benefit remains uncertain. This question should be examined in studies with larger samples or longer intervention durations. The protocol required only 20 min per session and minimal equipment, which may support implementation in both hospital and community settings. In clinical practice, LQG may be considered as an adjunct to standard rehabilitation for stroke survivors who are able to follow structured verbal and movement instructions, particularly when respiratory function and trunk-related deficits are both relevant treatment targets. The present findings also provide a preliminary basis for future studies to examine simplified or adapted LQG protocols in broader stroke populations, including patients with greater communication or cognitive limitations. Training tolerance and adverse events were monitored throughout the study, and no serious intervention-related adverse events were observed.

### Limitations and future directions

This study has several limitations. The intervention period was relatively short, and both groups received active standard rehabilitation. Under these conditions, the study may have been better suited to detect physiological changes than modest between-group differences in complex functional scales such as the BBS and TIS. This may partly explain why the additional gains observed in pulmonary function and sEMG were not paralleled by statistically significant between-group differences in these scale-based outcomes. In addition, EO sEMG provided only an indirect and superficial index of abdominal wall recruitment. It did not directly quantify deep core muscle activity or intra-abdominal pressure. The mechanistic interpretation of the present findings should therefore remain cautious. The exploratory path model offered statistical support for a hypothesized pathway, but it did not establish physiological causality. Likewise, higher-level explanations involving vocal tract mechanics, cortical motor networks, or autonomic regulation were not directly examined and remain speculative. Finally, this was a single-center study without long-term follow-up, which limits conclusions about the durability and generalizability of the observed effects. Future studies should include larger samples, longer intervention durations, direct physiological measurements, and longitudinal follow-up to determine whether the observed physiological gains can be translated into sustained clinical benefits in trunk control and balance.

## Conclusion

Adding LQG to standard rehabilitation yielded greater improvements in FVC, PEF, and affected-side EO activation after stroke. Although additional gains in trunk control and balance scales were not statistically significant over 3 weeks, exploratory analyses supported a possible respiratory-core-balance pathway that requires further validation. These findings suggest that LQG may serve as a useful adjunct to standard rehabilitation by enhancing respiratory and abdominal wall function, while its applicability in broader stroke populations and its translation into additional clinical gains in trunk control and balance remain to be confirmed.

## Data Availability

The original contributions presented in the study are included in the article and any published supplementary material, if applicable. Due to institutional policies and the terms of informed consent, the raw dataset is not publicly available. Further inquiries can be directed to the corresponding authors upon reasonable request.
